# The *PNPLA3* Genetic Variant rs738409 Influences the Progression to Cirrhosis in HIV/Hepatitis C Virus Coinfected Patients

**DOI:** 10.1371/journal.pone.0168265

**Published:** 2016-12-14

**Authors:** Rocío Núñez-Torres, Juan Macías, María Mancebo, Mario Frías, Giovanni Dolci, Francisco Téllez, Dolores Merino, Nicolás Merchante, Jesús Gómez-Mateos, Giovanni Guaraldi, Antonio Rivero-Juárez, Juan A. Pineda, Luis M. Real, on behalf of HEPAVIR-esteatosis Study Group

**Affiliations:** 1 Unit of Infectious Diseases and Microbiology, Valme University Hospital, Seville, Spain; 2 Instituto de Biomedicina de Sevilla (IBIS), Seville, Spain; 3 Unit of Infectious Diseases, Reina Sofía University Hospital, Córdoba, Spain; 4 Instituto Maimónides de Investigación Biomédica de Córdoba (IMIBC), University of Córdoba, Córdoba, Spain; 5 Department of Medical and Surgical Science for Adults and Children, Clinic and Infectious Diseases, University of Modena and Reggio Emilia, Modena, Italy; 6 Unit of Infectious Diseases, La Línea de la Concepción Hospital, Cádiz, Spain; 7 Unit of Infectious Diseases, Huelva University Hospital, Huelva, Spain; CAEBI, SPAIN

## Abstract

Contradictory data about the impact of the rs738409 steatosis-related polymorphism within *PNPLA3* gene on liver fibrosis progression in HIV/hepatitis C virus (HIV/HCV)-coinfected patients have been reported. Our objective was to test whether this, and other polymorphisms previously related to fatty liver disease in HIV infection linked to *SAMM50* or *LPPR4* genes, influence liver fibrosis progression in HIV/HCV-coinfected individuals. Three hundred and thirty two HIV/HCV-coinfected patients who consecutively attended four Spanish university hospitals from November 2011 to July 2013 were included. A liver stiffness cut-off of 14.6 kPa, as determined by transient elastography, was used to diagnose cirrhosis. Liver stiffness progression was studied in 171 individuals who had two available LS determinations without anti-HCV treatment between them. Moreover, 28 HIV/HCV-coinfected patients who underwent liver transplant, as well as 19 non-cirrhotic coinfected individuals used as controls, were included in an additional study. Only rs738409 was associated with cirrhosis: 45 (29.6%) of 152 G allele carriers versus 36 (20.0%) of 180 CC carriers showed cirrhosis (multivariate p = 0.018; adjusted odds ratio = 1.98; 95% confidence interval = 1.12–3.50). Also, 21 (30.4%) of 69 G allele carriers versus 16 (15.7%) of 102 CC patients showed significant liver stiffness progression (adjusted p-value = 0.015; adjusted odds ratio = 2.89; 95% confidence interval = 1.23–6.83). Finally, the proportion of rs738409 G allele carriers was significantly higher in transplanted individuals than in controls (p = 0.044, odds ratio = 3.43; 95% confidence interval = 1.01–11.70). Our results strongly suggest that the rs738409 polymorphism is associated with liver fibrosis progression in HIV/HCV-coinfected patients.

## Introduction

Chronic infection with hepatitis C virus (HCV) may lead to advanced liver fibrosis and end-stage liver disease. In HIV/HCV-coinfected individuals, liver fibrosis progression is accelerated [1, 2]. Therefore, these individuals show a higher frequency of cirrhosis and end-stage liver disease. In spite of this, the rate of liver fibrosis progression is variable between patients. Several risk factors for accelerated fibrosis have been previously identified in HIV/HCV-coinfected patients [2–7].

As in other clinical conditions, host genetic factors could also influence the variability in the progression of liver disease in HIV/HCV-coinfected patients. The identification of these factors could help to understand the molecular basis of liver disease in HIV/HCV-coinfected patients and may allow to identify those individuals at risk of developing advanced liver disease. Recently, several authors have analyzed whether the rs738409 genetic marker, located within the patatin-like phospholipase domain-containing 3 (*PNPLA3*) gene, could be a risk factor for liver fibrosis progression in HIV/HCV-coinfected patients with controversial results [8–11]. This single nucleotide polymorphism (SNP) has been consistently associated with fatty liver disease (FLD) in both HCV-uninfected and HCV-infected individuals [12, 13]. Similarly, other SNPs that have been recently associated with FLD in the HIV-infected population [14] could also have an impact on the faster liver fibrosis progression observed in the HIV/HCV-coinfected population, but this has not been explored so far.

Because of the above reasons, the aims of this study were: i) to test whether the *PNPLA3* rs738409 genetic marker is associated with liver damage progression in the HIV/HCV-coinfected population ii) to analyze if other SNPs, previously associated with FLD in HIV infected population, such as *SAMM50* rs738491 or *LPPR4* rs12743824, could also be related to this outcome.

## Patients and Methods

### Patients

This work comprises three different studies: two of them were cross-sectional (study 1 and study 3) and the other one was a longitudinal retrospective assessment (study 2). The study populations were as followed:

Study population 1. Those HIV-infected individuals with active HCV coinfection who consecutively attended the Infectious Diseases outpatient clinics in four university hospitals in Spain from November 2011 to July 2013 were included. All patients underwent a liver stiffness (LS) determination, as a noninvasive measurement of liver fibrosis, during a single visit previously scheduled as a routine follow-up visit. Data for this study were collected during the same visit. In all patients, a whole blood sample was collected for routine laboratory and genetic determinations.

From the entire study population 1, those patients who had two available LS determinations separated at least by one year, without having received any treatment against HCV infection between both determinations, were selected for the analysis of genetic associations with LS progression (LSP). Clinical and demographic data corresponding to the date of the first LS determination were obtained from the clinical records.

Study population 2. This population was composed of: i) Those HIV/HCV-coinfected patients who underwent cadaveric donor liver transplant because of end-stage liver cirrhosis related to HCV infection, and who donated genetic material at the time of transplantation, according to an established procedure of the Centro Nazionale Trapianti (http://www.trapianti.salute.gov.it/). All these individuals belonged to the Modena University Transplant cohort that started in 2003. ii) Those HIV/HCV-coinfected and non-cirrhotic individuals from the same geographical area who had a known date of coinfection and who had DNA samples available at the local bio-bank (Infectious Disease Clinic–Azienda policlinico Universitaria Modena, Italy).

All individuals were older than 18 and Caucasians. Pregnant women as well as those subjects who showed family relationship with other selected patients were excluded from all of the analyses.

### Transient elastography examination

Liver stiffness with controlled attenuation parameter (CAP) measurements were performed by an experienced operator at each participating center using a commercially available elastography device (FibroScan 502, Echosens, Paris, France). Elastography examinations were conducted as stated elsewhere [15, 16]. CAP is an estimate of the total ultrasonic attenuation and it is expressed in dB/m. LS is expressed in kilopascals (kPa). According to previously reported standards, examinations with 10 successful shots, an interquartile range for liver stiffness lower than 30% of the median value and a success rate of at least 60%, were considered as reliable [16]. Cut-off values of 238 dB/m and 14.6 kPa were selected to define the presence of significant steatosis and cirrhosis [15, 16], respectively.

### Significant liver stiffness progression and liver stiffness progression rate definitions

Significant LSP was defined as a 30% increment of LS in the second determination over the first LS determination, being the difference between both LS determinations higher than 3 kPa. The LSP rate (LSPR) was calculated as the differences in kPa between both determinations divided by the number of years elapsed between both determinations, and it was expressed in kPa/year.

### DNA isolation and polymorphism genotyping

DNA was extracted from frozen whole blood using the Quick Pure Blood DNA extraction Kit (Macherey-Nagel, Düren, Germany). The SNPs were genotyped using a custom Golden Gate protocol (Illumina, San Diego, California USA).

### Statistical analyses

The outcome variables were the presence of cirrhosis (study 1), the presence of significant LSP (study 2) and the diagnosis of end-stage liver disease (study 3).

The online resource at the Institute for Human Genetics, Munich, Germany (http://ihg.gsf.de) was used for testing comparison of genotypic frequencies between groups to determine different genetic models, p values, odds-ratios (OR), 95% confidence intervals (95% CI) as well as the Hardy-Weinberg equilibrium. As a reference for genotypic and allelic frequencies of rs738409 in the European Caucasian population, we used the data reported in the NCBI single nucleotide polymorphisms database (http://www.ncbi.nlm.nih.gov/projects/SNP/snp_ref.cgi?rs=738409).

To compare the categorical variables in two different groups of individuals the Pearson chi-square test was used. Quantitative variables among patient groups were compared by means of the Student´s t-test (data normally distributed) or Mann-Whitney U test (data not normally distributed). Logistic regression models were elaborated including variables with a univariate p-value <0.2, as well as age and gender to obtain adjusted p and OR values and to detect factors independently associated with the outcome variables. For the analysis of significant LSP, the time between LS determinations was included in the models along with age, gender and variables with an univariate p-value <0.2. All these analyses were carried out using the SPSS software 22.0 (IBM Corporation, Somers, NY, USA).

The estimation of power to detect alleles associated with cirrhosis was performed by the Episheet software available at http://members.aol.com/krothman.

### Ethic statement

This study was performed according to the ethical guidelines of the Declaration of Helsinki. The study was approved by the Ethics Committee of the Valme University Hospital and by the Provincial Ethics Committee of Modena (pratica 183/14). Written consent was obtained from all individuals before sampling.

## Results

### Study populations

Three hundred and thirty seven HIV-infected patients with active HCV coinfection were selected. Five (1.5%) of them had not a reliable LS determination and were excluded. Therefore, 332 individuals were included in the study population 1. Among them, a total of 124 (37.5%) individuals previously received treatment against HCV infection without sustained virological response (SVR). The main characteristics of this population are depicted in Table 1.

**Table 1:** Main characteristics of the study population 1 and the subpopulation where liver stiffness progression was evaluated.

Variables	Study population 1 (n = 332)	Subpopulation with two LS determinations * (n = 171)
Male gender, n (%)	287 (86.4)	145 (84.8)
Age^a^, years	47 (43–50)	45 (41–49)
Alcohol intake ≥ 50g/day, n (%)	59 (18.4) ^b^	38 (22.2) ^e^
BMI^a^, Kg/m2	23.5 (21.4–26.2) ^c^	22.8 (20.8–25.8) ^f^
Fasting plasma glucose^a^, mg/dL	94 (87–106)	94 (86.75–103)
Total plasma cholesterol^a^, mg/dL	166 (142.3–188.5)	167 (143.5–201.3)
Plasma LDL cholesterol^a^, mg/dL	91 (71–112)	93 (67–115)
Plasma HDL cholesterol^a^, mg/dL	43 (34–53)	40.5 (31–53)
Plasma tryglicerides^a^, mg/dL	135.5 (93.3–173.8)	144 (105.3–190.8)
ALT^a^, IU/L	50 (33–79)	52 (37–80)
AST^a^, IU/L	46 (31–69)	47 (34–72.5)
GGT^a^, IU/L	94 (55–174)	95 (53–175)
CD4^a^ cell/microL	479 (311–710)	469 (301–685)
Serum HIV-RNA <50 Copies/mL, n (%)	261 (78.6)	117 (70.1) ^g^
CDC stage C, n (%)	101 (30.7) ^d^	33 (29.2) ^h^
Current ART, n (%)	319 (96.1)	161 (94.2)
HCV Genotype, n (%)		
1	210 (63.3)	106 (62.0)
2	1 (0.3)	0 (0.0)
3	52 (15.7)	27 (15.8)
4	61 (18.4)	30 (17.5)
Unknown	8 (2.4)	8 (4.7)
HCV viral load^a^, log IU/mL	6.2 (5.6–6.6)	ND
Liver stiffness^a^, kPa	8.6 (5.8–13.8)	8.1 (5.8–13.2)
CAP^a^, dB/m	229.5 (201.2–260.8)	ND

LS, liver stiffness; BMI, body mass index; LDL, low-density lipoprotein; HDL, high-density lipoprotein; ALT, alanine aminotransferase; AST, aspartate aminotransferase; GGT, gamma-glutamyl transferase CDC, Centers for Disease Control and Prevention; ART, antiretroviral therapy; HCV, hepatitis C virus; CAP, controlled attenuation parameter; ND, not determined.

*values obtained at the date of the first Liver stiffness determination

^a^ Median (quartil 1 –quartil 3).

Available in

^b^320,

^c^309,

^d^329,

^e^157,

^f^80,

^g^167,

^h^113 patients.

To analyze LSP, one hundred and ninety-eight individuals who had two available LS determinations separated at least by one year were selected. However, 27 (13.6%) of them received anti-HCV treatment between LS determinations. Therefore, 171 (51.5%) out of 332 individuals from the study population 1 were included in this subpopulation. Among them, 24 (14.7%) had received anti-HCV treatment before the first LS determination without reaching SVR. The median (quartile 1 –quartile 3) period between both LS measurements was 2.20 (1.30–4.52) years. The median (quartile 1 –quartile 3) of LSPR was 0.06 (-0.50–1.10) kPa/year. The characteristics of this subpopulation are depicted in Table 1.

Regarding the study population 2, twenty-eight liver-transplanted individuals and 19 coinfected and non-cirrhotics individuals were included. The median (quartile 1 –quartile 3) age at the liver transplant date was 47.3 (43.8–50.2) years, and the median (quartile 1 –quartile 3) age of controls was 49.2 (45.1–53.6) years, (p = 0.29). The medians of time since the coinfection date were 22.3 (18.5–25.2) and 21.9 (10.0–27.5) years for liver transplanted and control individuals, respectively (p = 0.137). The gender distribution were also similar for both populations (p>0.400).

### Study 1. Genetic factors associated with cirrhosis

In the study population 1, cirrhosis was diagnosed in 81 (24.4%) individuals. The genotypic distributions of *SAMM50* rs738491, *LPPR4* rs12743824 and *PNPLA3* rs738409 polymorphisms in cirrhotic and non-cirrhotic individuals are displayed in Table 2. All of them were in accordance with the Hardy-Weinberg equilibrium (p>0.400 in all cases).

**Table 2:** Univariate and multivariate analysis of factors associated with cirrhosis (Study 1).

Variables	Univariate			Multivariate	
	Non-cirrhotics (n = 251)	Cirrhotics (n = 81)	p-value	AOR (95%CI)	Adjusted p-value
Male gender, n (%)	214 (85.3)	73 (90.1)	0.266	1.47 (0.64–3.41)	0.369
Age^a^, years	46 (43–50)	48 (45–51)	0.006	1.12 (1.06–1.19)	<0.001
Alcohol intake ≥ 50g/day, n (%)	38 (15.8)	21 (26.6)	0.031	1.56 (0.78–3.13)	0.208
BMI^a^, Kg/m2	23.7 (21.5–26.2)	23.2 (21.4–26.2)	0.852	NI	NI
Fasting plasma glucose^a^, mg/dL	94 (87–105)	96 (86–112)	0.218	NI	NI
Plasma total cholesterol^a^, mg/dL	171 (150–190)	150 (121–175)	<0.001	0.981 (0.97–0.98)	<0.001
Plasma Triglycerides^a^, mg/dL	134 (93–176)	136 (92–160)	0.827	NI	NI
HIV-RNA <50 Copies/mL, n (%)	200 (79.7)	61 (75.3)	0.404	NI	NI
CDC stage C, n (%)	76 (30.5)	25 (31.2)	0.902	NI	NI
Current ART, n (%)	243 (96.8)	76 (93.8)	0.228	NI	NI
Significant steatosis, n (%)	124 (39.1)	44 (45.4)	0.273	NI	NI
HCV Genotype 3, n (%)	42 (17.1)	10 (12.7)	0.352	NI	NI
HCV viral load, Log (IU/mL) ^a^	6.2 (5.6–6.7)	6.0 (5.4–6.5)	0.051	0.67 (0.46–0.97)	0.035
rs738491 (*SAMM50*)			0.527^b^	NI	NI
CC, n (%)	129 (51.4)	36 (44.4)			
CT, n (%)	98 (39.0)	35 (43.2)			
TT, n (%)	24 (9.6)	10 (12.3)			
rs12743824 (*LPPR4*)			0.513^b^	NI	NI
AA, n (%)	54 (21.5)	13 (16.0)			
AC, n (%)	117 (46.6)	39 (48.1)			
CC, n (%)	80 (31.9)	29 (35.8)			
rs738409 (*PNPLA3*)			0.042^b^	1.98 (1.12–3.50)	0.018
CC, n (%)	144 (57.4)	36 (44.4)			
GC, n (%)	90 (35.9)	35 (43.2)			
GG, n (%)	17 (6.8)	10 (12.3)			

BMI, body mass index; CDC, Centers for Disease Control and Prevention; ART, antiretroviral therapy; PI, Protease Inhibitor; HCV, hepatitis C virus; *SAMM50*, sorting and assembly machinery component 50 homolog; *LPPR4*, lipid phosphate phosphatase-related protein type 4; *PNPLA3*, patatin-like phospholipase domain containing 3; CI, confidence interval; AOR, adjusted odds ratio; NI, not included in the model.

^a^ Median (interquartile range).

^b^ Pearson Chi-square (TT vs. CC+CT for rs738491, AA+AC vs. CC for rs12743824, GG+GC vs. CC for r rs738409).

Only the *PNPLA3* rs738409 genetic marker was associated with cirrhosis. Thus, we observed that among 152 individuals carrying the G allele (GG and GC genotypes), 45 (29.6%) showed this condition, whereas the proportion of cirrhotic subjects among those bearing the CC genotype was 20.0% (36 out of 180 individuals) (p = 0.042; OR = 1.683; 95%CI = 1.016–2.786).

In the univariate analysis, other factors associated with cirrhosis were older age, lower total cholesterol levels and alcohol intake (Table 2). Factors that remained independently related to cirrhosis in the multivariate analysis were the presence of the *PNPLA3* rs738409 G allele, older age, lower HCV viral load and total cholesterol levels (Table 2).

### Study 2. Factors associated with significant liver stiffness progression

Among the 171 patients with paired LS determinations, 37 (21.6%) were classified as individuals with significant LSP. No differences were observed in the median time between LS determinations in patients with and without significant LSP (median [quartile 1 –quartile 3]: 2.56 [1.27–5.39] years versus 2.17 [1.29–4.30] years, respectively; p = 0.561).

The genotypic distributions of the three SNPs analyzed are displayed in Table 3.

**Table 3:** Associations with significant liver stiffness progression (Study 2).

Variables	Univariate			Multivariate	
	Non-significant LSP (n = 134)	Significant LSP (n = 37)	p-value	AOR (95%CI)	Adjusted p-value
Male gender, n (%)	112 (83.6)	33 (89.2)	0.605	1.41 (0.392–5.04)	0.599
Age^a^, years	44 (40–49)	46 (42–49)	0.652	1.04 (0.96–1.12)	0.307
Alcohol intake ≥ 50g/day, n (%)	26 (20.8)	12 (37.5)	0.049	2.63 (1.04–6.67)	0.041
BMI^a^, Kg/m2	23.1 (20.8–26.1)	22.1 (20.5–23.8)	0.827	NI	NI
Plasma glucose^a^, mg/dL	94 (87–103)	95 (85–104)	0.911	NI	NI
Plasma total cholesterol^a^, mg/dl	171 (146–202.5)	160 (138.5–185.5)	0.533	NI	NI
Plasma HDL cholesterol^a^, mg/dl	40 (31–53)	41 (31–53)	0.781	NI	NI
Plasma tryglicerides^a^, mg/dl	145 (108–188)	143 (88–197)	0.944	NI	NI
ALT^a^, IU/L	49 (37–78)	61 (36–119)	0.094	0.99 (0.98–1.01)	0.849
AST^a^, IU/L	44 (33.5–67)	58 (41–111.5)	0.015	1.01 (0.99–1.01)	0.331
GGT^a^, IU/L	91 (52–160)	138 (57.5–222)	0.048	1.00 (0.99–1.01)	0.051
HIV-RNA <50 Copies/mL, n (%)	94 (71.2)	23 (65.7)	0.528	NI	NI
CDC stage C, n (%)	26 (29.9)	7 (26.9)	0.771	NI	NI
Current ART, n (%)	128 (95.5)	33 (89.2)	0.226	NI	NI
HCV Genotype 3, n (%)	21 (16.5)	6 (16.7)	0.985	NI	NI
Time between LS determinations^a^, years	2.2 (1.3–4.3)	2.5 (1.3–5.4)	0.561	1.08 (0.88–1.33)	0.844
Cirrhosis at baseline, n (%)	28 (20.9)	10 (27.0)	0.427	NI	NI
rs738491 (*SAMM50*)			0.577^b^	NI	NI
CC, n (%)	76 (56.7)	18 (48.6)			
CT, n (%)	41 (30.6)	13 (35.1)			
TT, n (%)	17 (12.7)	6 (16.2)			
rs12743824 (*LPPR4*)			0.569^b^	NI	NI
AA, n (%)	24 (17.9)	7 (18.9)			
AC, n (%)	66 (49.3)	16 (43.2)			
CC, n (%)	44 (32.8)	14 (37.8)			
rs738409 (*PNPLA3*)			0.022^b^	2.89 (1.23–6.83)	0.015
CC, n (%)	86 (64.2)	16 (43.2)			
GC, n (%)	38 (28.4)	18 (48.2)			
GG, n (%)	10 (7.5)	3 (8.1)			

LSP, Liver stiffness progression; BMI, body mass index; ALT, alanine aminotransferase; AST, aspartate aminotransferase; GGT, gamma glutamyl transpeptidase; CDC, Centers for Disease Control and Prevention; ART, antiretroviral therapy; HCV, hepatitis C virus; *SAMM50*, sorting and assembly machinery component 50 homolog; *LPPR4*, lipid phosphate phosphatase-related protein type 4; *PNPLA3*, patatin-like phospholipase domain containing 3; CI, confidence interval; AOR, adjusted odds ratio; NI, not included in the model.

^a^ Median (interquartile range).

^b^ Pearson Chi-square (TT vs. CC+CT for rs738491, AA+AC vs. CC for rs12743824, GG+GC vs. CC for r rs738409).

**Fig 1:**
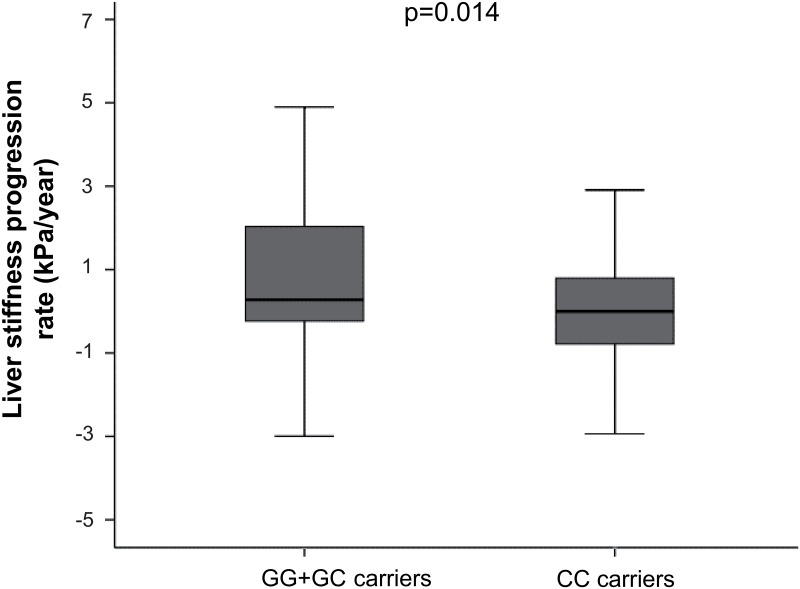
Liver stiffness progression rate according to *PNPLA3* rs738409 genotype.

No association was found between *SAMM50* rs738491 or *LPPR4* rs12743824 and significant LSP. A higher proportion of *PNPLA3* rs738409 G allele carriers was observed in individuals with significant LSP. Thus, among 69 individuals carrying the G allele, 21 (30.4%) showed significant LSP, whereas for those bearing the CC genotype the frequency of significant LSP was 15.7% (16 out of 102 individuals) (p = 0.022; OR = 2.35; 95% CI = 1.12–4.92). Accordingly, LSPR was significantly higher in GG/GC carriers than that observed for CC carriers (median [quartile 1 –quartile 3]: 0.27 [-0.25–2.07] kPa/year versus 0.00 [-0.78–0.81] kPa/year, respectively; p = 0.014) (Fig 1).

Table 3 shows other factors associated with significant LSP in the univariate analysis. In the multivariate analysis, only the presence of the *PNPLA3* rs738409 G allele and high alcohol intake remained as independent factors associated with this outcome (Table 3). When the same analysis was carried out excluding individuals with cirrhosis at baseline the presence of the *PNPLA3* rs738409 G showed a trend to association with significant LSP (adjusted p-value = 0.053; adjusted odds ratio = 2.73; 95% CI = 0.98–7.56).

### Study 3. Role of rs738409 in patients with end stage of liver disease

We analyzed the *PNPLA3* rs738409 genotypic distribution in 28 HIV/HCV-coinfected individuals who underwent a liver transplant because of end-stage liver disease, as well as, in 19 coinfected and non-cirrhotics controls (study population 2). In the end-stage liver disease group, 8 (28.6%) individuals were CC, 16 (57.1%) were GC and 4 (14.3%) were GG. The corresponding figures for the control group were 11 (57.9%) CC, 7 (36.8%) GC and 1 (5.3%) GG. The genotypic distribution and the allelic frequencies of these polymorphisms observed in the control group were in accordance with those published for European Caucasian populations (p>0.700). The proportion of GG/GC carriers was higher in HIV/HCV-coinfected patients with end-stage liver disease than in control individuals (p = 0.044, OR = 3.43; CI = 1.01–11.70).

## Discussion

The results obtained in this study support that the FLD-related *PNPLA3* rs738409 genetic variant is associated with liver disease progression in HIV/HCV-coinfected patients. On the contrary, others SNPs associated with FLD in the HIV-infected population such as *SAMM50* rs738491 and *LPPR4* rs12743824 do not seem to have a relevant role on liver disease evolution in these patients.

Even though *PNPLA3* rs738409 has been widely studied in different chronic liver diseases, the influence of this polymorphism on liver fibrosis progression in HIV/HCV-coinfected patients has not been consistently established [8–11]. Thus, Mandorfer et al, [11] identified an univariate association between *PNPLA3* rs738409 G allele and faster fibrosis progression rate in a preliminary study carried out in a small sample of HIV/HCV-coinfected individuals. However, they did not detected any relationship between *PNPLA3* rs738409 and stage of fibrosis in a recent study performed in 177 HIV/HCV-coinfected patients [9]. Similarly, the authors of a recent work analyzed 168 coinfected patients and reported no association between this variant and advanced liver fibrosis [10]. By contrast, in another study performed in a sample of 215 HIV/HCV-coinfected individuals, a significant association between *PNPLA3* rs738409 G allele and advanced fibrosis was found [8]. Nevertheless, in the latter study, the association of the *PNPLA3* genetic variation with fibrosis progression did not reach the level of statistical significance. Since the development of advanced fibrosis in HCV-infected individuals is influenced by the duration of HCV infection, and, consequently, by age [17], the contradictory results about the association between *PNPLA3* rs738409 G allele and fibrosis could be due to the cross-sectional design of some of these studies. The size of the sample included is an additional issue. Thus, association studies with advanced liver fibrosis as end-point need large populations and/or validations in separate populations. These requirements were not fulfilled in the above-stated studies. A more comprehensive approach to this problem is to test the influence of *PNPLA3* rs738409 G allele on the progression of liver disease. Two studies analyzed this outcome and reported a trend toward the association [8] or an univariate association [11]. Our study, which was carried out in a larger sample, identified the presence of *PNPLA3* rs738409 G allele as an independent risk factor for cirrhosis in HIV/HCV-coinfected patients. Moreover, as a validation of this finding, we detected a higher frequency of *PNPLA3* rs738409 G allele carriers in a group of HIV-infected individuals who had reached end-stage of liver disease due to HCV infection. This association was also confirmed by proving a relationship between this polymorphism and significant LSP. Taken together, our results clearly support the implication of *PNPLA3* rs738409 on fibrosis progression and, ultimately, the development of cirrhosis in HIV/HCV coinfection. These findings are also in agreement with those obtained in the HCV monoinfection setting where *PNPLA3* rs738409 has been associated with both, cirrhosis [18, 19] and fibrosis progression [20–22].

We previously reported a lack of association between the *PNPLA3* rs738409 variant and FLD in HIV/HCV-coinfected individuals [14]. Although Signelli et al. found a relation of this SNP with steatosis in a small population [10], our finding has been replicated by others [8, 9]. This was a relatively surprising result since this genetic variant has been extensively related to non-alcoholic FLD in general population [23–25] and in HCV-monoinfected patients [18, 20, 26, 27]. The relation of this genetic variant to cirrhosis without a clear association with FLD in the HIV/HCV-coinfected population could be pointing out to the existence of other factors that modulate the effect of this variant on the liver [22]. More studies are warranted to elucidate this issue. By contrast, the other SNPs evaluated here, linked to *SAMM50* and *LPPR4* genes, which were previously associated with FLD in the HIV-infected population [14], have not shown any association with cirrhosis or significant LSP in our study. Taking into account the sample size analyzed, the case-control ratio and the minor allele frequency of those genetic markers in our population, our study had a 75% and 80% power to detect OR = 2 for the study of association of *SAMM50* rs738491 and *LPPR4* rs12743824 polymorphisms with cirrhosis, respectively. Consequently, the absence of association with cirrhosis is probably not due to the lack of statistical power. Therefore, our results suggest that these genetic markers do not have a prevalent role on fibrosis progression.

During the last years, many studies have focused on unraveling the pathophysiological mechanisms of PNPLA3 effects. However they are still unclear [28]. PNPLA3 is a lipase highly expressed in human liver and adipose tissue. Alterations produced by *PNPLA3* rs738409 polymorphism yield changes in the enzyme catalytic binding site leading to the cellular triglyceride accumulation [29]. This mechanism could explain the etiology of FLD and its relation to *PNPLA3* rs738409 polymorphism. However, this hypothesis have not been validated in any *in vivo* experiment performed so far [28]. In addition, PNPLA3 also seems to have a key role as a liver metabolic modulator, and *PNPLA3* rs738409 polymorphism could produce a shift to anaerobic metabolism and mitochondrial dysfunction in hepatocytes [30]. This could explain its contribution to other liver diseases, as HCV-related liver disease. However, more studies are necessary to test this hypothesis.

Our study has some limitations. First, the sample sizes of study population 2 and the subpopulation where LSP was analyzed, were relatively small. In spite of this, the effect of *PNPLA3* rs738409 was in the same direction in all cases. Second, the longitudinal analysis carried out in that subpopulation was retrospective. Therefore, the quality and quantity of clinical data could have been limited to their availability and, selection bias cannot be excluded. Nevertheless, we did not observe considerable differences between the study population 1 and the subpopulation where LSP was analyzed (Table 1). Third, we included cirrhotic patients at the date of the first LS determination (baseline) to assess factors associated with significant LSP. Generally, these patients are excluded when fibrosis progression is evaluated in liver biopsy studies. This was due to the calculation of fibrosis progression using fibrosis staging classifications, where individuals in the last stage of fibrosis are not susceptible to progress anymore. However, LSP also occurs in cirrhosis stage (>14.5 kPa) and, it has been reported that patients with LS values over specific LS cut-off values, such as 21 kPa or 40 kPa, have a poorer prognosis [31, 32]. In addition, increases in LS in patients with cirrhosis have been proven to be associated with liver decompensation [33, 34]. Thus, we believe that the inclusion of these individuals in the study of LSP allows a comprehensive analysis of this trait. Of note, when these individuals were not included, the presence of the *PNPLA3* rs738409 G allele trended to be associated with significant LSP. Fourth, since we used significant LSP as the main variable instead of LSPR, the associations found with significant LSP might be confounded by the length of time between LS determinations. For that reason, multivariate analysis performed in the subpopulation where LSP was studied was adjusted by this variable. Moreover, it is important to note that LSPR was higher in those patients bearing the *PNPLA3* rs738409 G allele (Fig 1).

In conclusion, our results, in combination with previous evidence, confirm a role of *PNPLA3* rs738409 in fibrosis progression in HIV/HCV co-infection. Because of the effect of this polymorphism on LSP (Table 3), this genetic marker should be taken into account, together with other risk factors, for identifying coinfected patients at high likelihood of developing advanced fibrosis. Hence, it could be useful for the prioritization of those patients who should receive anti-HCV treatment with direct-acting antivirals, mainly in those countries where the use of these antivirals is restricted.
